# An Investigational Study on the Role of *CYP2D6*, *CYP3A4* and *UGT*s Genetic Variation on Fesoterodine Pharmacokinetics in Young Healthy Volunteers

**DOI:** 10.3390/ph17091236

**Published:** 2024-09-19

**Authors:** Andrea Rodríguez-Lopez, Dolores Ochoa, Paula Soria-Chacartegui, Samuel Martín-Vilchez, Marcos Navares-Gómez, Eva González-Iglesias, Sergio Luquero-Bueno, Manuel Román, Gina Mejía-Abril, Francisco Abad-Santos

**Affiliations:** 1 Clinical Pharmacology Department, Hospital Universitario de La Princesa, Faculty of Medicine, Universidad Autónoma de Madrid (UAM), Instituto de Investigación Sanitaria La Princesa (IP), 28006 Madrid, Spain; arodriguezl.externo@salud.madrid.org (A.R.-L.); mdolores.ochoa@salud.madrid.org (D.O.); paula.soria.externo@salud.madrid.org (P.S.-C.); smvilchez@salud.madrid.org (S.M.-V.); marcos.navares@salud.madrid.org (M.N.-G.); egiglesias@salud.madrid.org (E.G.-I.); manuel.roman@salud.madrid.org (M.R.); ginapaola.mejia@salud.madrid.org (G.M.-A.); 2 Centro de Investigación Biomédica en Red de Enfermedades Hepáticas y Digestivas (CIBERehd), Instituto de Salud Carlos III, 28029 Madrid, Spain

**Keywords:** fesoterodine, pharmacogenetics, *CYP2D6*

## Abstract

***Introduction:*** Fesoterodine is one of the most widely used antimuscarinic drugs to treat an overactive bladder. Fesoterodine is extensively hydrolyzed by esterases to 5-hydroxymethyl tolterodine (5-HMT), the major active metabolite. CYP2D6 and CYP3A4 mainly metabolize 5-HMT and are, therefore, the primary pharmacogenetic candidate biomarkers. ***Materials and Methods:*** This is a candidate gene study designed to investigate the effects of 120 polymorphisms in 33 genes (including the CYP, COMT, UGT, NAT2, and CES enzymes, ABC and SLC transporters, and 5-HT receptors) on fesoterodine pharmacokinetics and their safety in 39 healthy volunteers from three bioequivalence trials. ***Results:*** An association between 5-HMT exposure (dose/weight corrected area under the curve (AUC/DW) and dose/weight corrected maximum plasma concentration (C_max_/DW)), elimination (terminal half-life (T_1/2_) and the total drug clearance adjusted for bioavailability (Cl/F)), and CYP2D6 activity was observed. Poor/intermediate metabolizers (PMs/IMs) had higher 5-HMT AUC/DW (1.5-fold) and C_max_/DW (1.4-fold) values than the normal metabolizers (NMs); in addition, the normal metabolizers (NMs) had higher 5-HMT AUC/DW (1.7-fold) and C_max_/DW (1.3-fold) values than the ultrarapid metabolizers (UMs). Lower 5-HMT exposure and higher T_1/2_ were observed for the CYP3A4 IMs compared to the NMs, contrary to our expectations. ***Conclusions:*** CYP2D6 might have a more important role than CYP3A4 in fesoterodine pharmacokinetics, and its phenotype might be a better predictor of variation in its pharmacokinetics. An association was observed between different genetic variants of different genes of the *UGT* family and AUC, C_max_, and CL/F of 5-HMT, which should be confirmed in other studies.

## 1. Introduction

Fesoterodine is a specific competitive muscarinic receptor antagonist used to treat an overactive bladder (OAB). An OAB is a chronic and frequently debilitating condition, distinguished by the symptom of urgency, which may or may not be associated with urinary incontinence and is often accompanied by an increased frequency of urination and nocturia [1]. Patients with an overactive bladder (OAB) are frequently underdiagnosed and undertreated. In a study of a Spanish population aged 60 years and older, 50% of participants regarded their OAB symptoms as typical for their age or sex, which may contribute to the significant delay in seeking medical attention [2]. The perception that incontinence is a normal part of aging, coupled with a lack of awareness that it is a treatable condition, may hinder access to diagnosis and treatment [3]. In clinical practice, managing older patients with OAB symptoms can be challenging due to the presence of multiple chronic comorbidities, concurrent medications, and functional or cognitive impairments [4]. OAB treatment should be tailored to each individual, considering the patient’s expectations, tolerability, the absence of drug–drug interactions, and cognitive safety [5].

The first-line treatment for an overactive bladder is based on behavioral therapies, lifestyle changes, and medical treatments. In terms of medication, the most commonly used treatments are antimuscarinics, such as fesoterodine [6]. Fesoterodine was developed from tolterodine and has shown superior efficacy to extended-release tolterodine [7] and to placebos [1]. Fesoterodine, along with dutasteride and finasteride, was classified as B (beneficial) by the low urinary tract symptoms fit-for-the-aged guidelines (LUTS-FORTA 2014), indicating that it is a medicinal product with proven efficacy in the elderly, with limited side effects and/or safety concerns [8].

Serving as a prodrug, fesoterodine is rapidly and extensively converted into 5-HMT without undergoing initial hepatic activation. Instead, it is metabolized by non-specific ubiquitous peripheral esterases, which are unaffected by age. A dose adjustment is unnecessary in cases of mild or moderate hepatic dysfunction or renal impairment, although caution should be exercised when increasing the dose. However, fesoterodine is contraindicated in severe hepatic impairment or concomitant treatment with potent CYP3A4 inducers [9,10,11]. In older patients, no dosage adjustment is required for fesoterodine, and food intake does not significantly impact its pharmacokinetics. The extended-release formulation enables once-daily administration for both 4 mg and 8 mg doses, facilitating flexible dosing and promoting adherence [9,12].

Fesoterodine is administered orally and is rapidly hydrolyzed by non-specific plasma esterases to 5-hydroxymethyl tolterodine (5-HMT), the major active metabolite and the main pharmacological agent. No parent drug has been observed in plasma after administration. The bioavailability of the active metabolite is 52%, and its plasma concentrations are proportional to the dose received, reaching maximum plasma levels after 5 h (T_max_). There is no accumulation after administration of multiple doses. 5-HMT is metabolized in the liver by CYP2D6 and CYP3A4 to carboxy-N-desipropyl and N-desipropyl, neither of which have antimuscarinic activity. The terminal half-life (T_1/2_) of 5-HMT is about 7 h and is not affected by the CYP2D6 phenotype [9].

In terms of adverse drug reactions (ADRs), the only one described as very frequent is dry mouth (>1/10). Frequent ADRs (≥1/100 to <1/10) include insomnia, dizziness, headache, dry eye, dry throat, abdominal pain, diarrhea, dyspepsia, constipation, nausea, and dysuria [9].

Given the importance of the disease, its impact on a patient’s quality of life, and the fact that fesoterodine is one of the most widely used antimuscarinic drugs, a candidate gene study was proposed to search for pharmacogenetic biomarkers that help to increase the efficacy in non-responders or to avoid side effects in responders.

## 2. Results

### 2.1. Demographic Characteristics

This study included a total of 39 volunteers. Weights, heights, and BMIs were lower in the women compared to the men (*p*_uv_ < 0.001, *p*_uv_ < 0.001, and *p*_uv_ = 0.031, respectively). Europeans presented with a lower age than Latin Americans or Sub-Saharan Africans (*p*_uv_ < 0.002). One volunteer was Sub-Saharan African, who was included with the Latin Americans in a new group called ‘Other’. The volunteers in clinical trial A were younger than the volunteers in clinical trial B (*p*_uv_ = 0.013) (Table 1).

### 2.2. Pharmacokinetics

The area under the curve (AUC) and the maximum plasma concentration (C_max_) were similar in the women and men after a dose/weight (DW) correction (Table 2). The women showed a lower T_1/2_ compared to the men (*p*_uv_ = 0.007; β = 1.262, R^2^ =0.459, *p*_mv_ < 0.001), with no significant differences in the T_max_ and total drug clearance adjusted for bioavailability (Cl/F) (Table 2).

No significant differences in the AUC/DW, C_max_/DW, T_max_, T_1/2,_ and Cl/F were observed when considering the different biogeographic origins (Table 2).

The AUC and C_max_ were lower in clinical trial A (4 mg single dose) compared to clinical trials B (8 mg single dose) and C (8 mg multiple dose) (19.29 ± 3.59 vs. 44.35 ± 16.47 h*ng/mL, *p*_uv_ = 0.012, and 57.84 ± 24.50 h*ng/mL, *p*_uv_ = 0.001; and 1.73 ± 0.22 vs. 4.98 ± 1.38 ng/mL, *p*_uv_ < 0.001, and 5.56 ± 1.94 ng/mL, *p*_uv_ < 0.001, respectively). However, no significant differences between clinical trials were observed in the AUC after adjusting by the DW (Table 2), whereas the C_max_/DW remained lower in clinical trial A compared to clinical trial C (*p*_uv_ = 0.023). T_1/2_ was significantly lower in clinical trial B compared to clinical trial C (*p*_uv_ < 0.001) (Table 2).

According to the food conditions, T_1/2_ was significantly lower in the fed volunteers compared to the fasting volunteers (*p*_uv_ < 0.001, β = −1.794, R^2^ = 0.459, *p*_mv_ = 0.003), with no significant differences in the AUC/DW, C_max_/DW, T_max_, and Cl/F (Table 2).

Regarding the genetic analysis, subjects with the *CES1* rs2244613 C/A genotype were associated with a lower T_1/2_ compared to those with the A/A genotype (*p*_uv_ = 0.026) (Table 3). However, this association was not maintained in the multivariate analysis.

For the analysis of CYP2D6 phenotypes, IMs (*n* = 12) and PMs (*n* = 1) were pooled. The AUC/DW was significantly lower in the CYP2D6 UMs compared to the NMs (*p*_uv_ = 0.008) and IMs/PMs (*p*_uv_ < 0.001; β = 0.130, R^2^ = 0.410, *p*_mv_ = 0.001) and in the CYP2D6 NMs compared to the IMs/PMs (*p*_uv_ = 0.005). The CYP2D6 UMs and NMs showed a lower C_max_/DW than the IMs/PMs (*p*_uv_ = 0.006 and *p*_uv_ = 0.002, respectively; β = 4.398, R^2^ = 0.461, *p*_mv_ = 0.002). T_1/2_ was significantly lower in the CYP2D6 UMs compared to the NMs and IM/PMs (*p*_uv_ = 0.015 and *p*_uv_ = 0.025, respectively). Lastly, Cl/F was significantly higher in the CYP2D6 UMs compared to the NMs and IMs/PMs (*p*_uv_ = 0.008 and *p*_uv_ < 0.001, respectively; β = −0.131, R^2^ =0.409, *p*_mv_ = 0.001) and lower in the CYP2D6 IMs/PMs compared to the NMs (*p*_uv_ = 0.005) (Table 3).

The CYP3A4 IMs showed a lower AUC/DW (*p*_uv_ = 0.046; β = −0.272, R^2^ =0.410, *p*_mv_ = 0.047) and higher CL/F (*p*_uv_ = 0.044; β = 0.275, R^2^ =0.409, *p*_mv_ = 0.045) than the NMs (Table 3).

The *UGT1A* rs10929302 A/A genotype was associated with a lower AUC/DW compared to the G/A and G/G genotypes (*p*_uv_ = 0.05 and *p*_uv_ = 0.024, respectively; β = −0.441, R^2^ =0.410, *p*_mv_ = 0.029) and a higher Cl/F compared to the G/A genotype (*p*_uv_ = 0.027; β = 0.436, R^2^ =0.409, *p*_mv_ = 0.031). The *UGT1A3/4* rs2008584 A/A genotype, the *UGT1A4* rs2011425 T/T genotype, and the *UGT2B7* rs7668258 C/C genotype were associated with a higher C_max_/DW compared to the *UGT1A3/4* rs2008584 A/G genotype (*p*_uv_ = 0.048; β = −0.881, R^2^ =0.461, *p*_mv_ = 0.023), *UGT1A4* rs2011425 T/G genotype (*p*_uv_ = 0.048) and *UGT2B7* rs7668258 T/C genotype (*p*_uv_ = 0.023; β = 10.111, R^2^ =0.461, *p*_mv_ = 0.011), respectively. Additionally, subjects with the *UGT2B7* rs7668258 T/C genotype were associated with a higher T_max_ compared to those with the *UGT2B7* rs7668258 T/T and C/C genotypes (*p*_uv_ = 0.035 and *p*_uv_ = 0.007, respectively) (Table 3).

No differences or trends in the pharmacokinetic parameters were observed when considering the remaining genes of metabolism, transport, or 5-HT receptors (Supplementary Table S1).

### 2.3. Safety

A total of 12 of the 39 healthy volunteers (78 exposures since each volunteer was exposed twice) experienced at least one ADR for a total of 30 ADRs. The most common ADR was headache (43.33%), followed by dry mouth (36.67%). The ADRs, such as nasal dryness, gastrointestinal motility, asthenia, palpitations, constipation, and dry skin, were reported only once.

No significant differences were observed in the incidence of ADRs according to sex, origin biogeographic, clinical trial, food conditions, genotype, haplotype, or phenotype. No significant differences were observed for the AUC (49.28 ± 19.26 vs. 41.58 ± 21.87 h*ng/mL, *p*_uv_ = 0.30), C_max_ (5.29 ± 2.03 vs. 4.34 ± 1.81 ng/mL, *p*_uv_ = 0.151), AUC/DW, C_max_/DW, T_max_, T_1/2_, or CL/F between the volunteers with ADRs and volunteers without ADRs.

## 3. Discussion

The pharmacokinetics of fesoterodine are modified by *CYP2D6* polymorphisms, suggesting that this enzyme is the main enzyme involved in its metabolism, which could also affect the efficacy of the drug.

Several studies with fesoterodine have shown efficacy in patients aged ≥65 years with an OAB. These data contribute to the LUTS-FORTA classification of fesoterodine as “beneficial”. Tolerability in these medically complex and vulnerable patients could be extended to all OAB patients, offering the benefits of flexible dosing and allowing an individualized risk–benefit balance according to the patient’s preference [3]. Based on the available data on fesoterodine and its advantages over other OAB drugs, further studies are needed to improve its prescribing and tolerability.

The fesoterodine drug label indicates that the plasma concentrations of its active metabolite are dose-proportional after single or multiple doses of fesoterodine, ranging from 4 mg to 28 mg, which is consistent with our results: the AUC in clinical trial B (dose: 8 mg) was twice the AUC in clinical trial A (dose: 4 mg) [9,11]. Additionally, the T_max_ and T_1/2_ observed in our study were similar to those included in the drug label (T_max_ = 5 h, T_1/2_ = 7 h) [9,11]. In this research, no significant differences in the pharmacokinetic parameters were observed between the men and women, except for T_1/2_, which was lower in the women. These results partially agree with those of the fesoterodine drug label, in which no differences in fesoterodine pharmacokinetics are described according to sex [9,11]. The lower T_1/2_ observed in women might be caused by the biological differences with men, such as the difference in body fat percentage, or genetic differences, such as an increased expression of enzymes like CYP3A4, which are involved in fesoterodine metabolism [13]. It is also noted that fesoterodine can be taken with or without food since food was not found to clinically alter fesoterodine pharmacokinetics [9,11]. In our case, there were no significant differences in the pharmacokinetic parameters depending on the feeding conditions, except for T_1/2_, where a decrease was observed, but since exposure did not change, the variation in T_1/2_ may be considered to be of no clinical relevance.

The active metabolite of fesoterodine, 5-HMT, is metabolized by CYP2D6 and CYP3A4 [9,11]. In this research, an association between 5-HMT exposure (AUC/DW and C_max_/DW) and elimination (T_1/2_ and Cl/F) and CYP2D6 activity was observed. The PMs/IMs had higher 5-HMT AUC/DW (1.5-fold) and C_max_/DW (1.4-fold) values than the NMs; in addition, the NMs had higher 5-HMT AUC/DW (1.7-fold) and C_max_/DW (1.3-fold) values than the UMs. The drug label indicates that the mean C_max_ and AUC values of the active metabolite are 1.7-fold and 2-fold higher, respectively, in the CYP2D6 PMs compared to the NMs. These differences were also observed in this study, although it should be noted that the CYP2D6 IMs and PMs subjects had to be pooled for analysis due to the small sample size. Furthermore, higher AUC mean values in the CYP2D6 IMs/PMs than in the UMs were also observed. These results clearly show that *CYP2D6* affects the pharmacokinetics of fesoterodine, but further studies are needed to evaluate whether these differences are clinically relevant.

Additionally, lower 5-HMT exposure and higher T_1/2_ were observed for the CYP3A4 IMs compared to the NMs. Since CYP3A4 is involved in 5-HMT metabolism for its elimination, a higher drug exposure should be expected in individuals with lower enzyme activity, which is contrary to our results. *CYP3A4* is a highly inducible gene, in which a higher hepatic activity in women compared to men was described [13], and the frequency in women was higher between the CYP3A4 IMs (42%) than between the CYP3A4 NMs (34%) in this research. Also, a variation in its expression levels was observed between different biogeographic origins, independently of the genotypes [14]. Also, higher CYP2D6 activity in the CYP3A4 IMs (the CYP2D6 UMs and NMs represented 71.4% of the CYP3A4 IMs, compared to 65.4% of the CYP3A4 NMs) was observed in this research. It has been difficult to evaluate the effect of *CYP3A4* on the pharmacokinetics of fesoterodine (with the data available to us) because, in this research, any PM has not been found, and CYP3A4 is also an enzyme that is easily induced or inhibited by various substances, including food. Thus, it could be proposed that CYP2D6 might have a more important role than CYP3A4 in fesoterodine pharmacokinetics, and its phenotype might be a better predictor of variation in its pharmacokinetics. Further studies with larger sample sizes and a better representation of all types of CYP3A4 metabolizer phenotypes would be needed to conclude its relevance as a pharmacogenetic biomarker.

In this research, an association was observed between the different genetic variants of different genes of the *UGT* family and some pharmacokinetic parameters of fesoterodine, such as AUC, C_max_, and CL/F. The *UGT*s are not listed on fesoterodine drug labels, and to our knowledge, no association between the *UGT*s and fesoterodine has been reported in other studies. The *UGT* genes are not completely characterized, the impact of its genetic variation is not clearly known, and no alleles have been defined, so further studies are needed to better characterize these genes and analyze their role in fesoterodine pharmacokinetics.

### Study Limitations

The main limitation of this study was the modest sample size, which reduced the statistical power and the chances of encountering biomarkers of interest with low prevalence within the sample population (e.g., CYP2D6 UM and PM, CYP3A4 IM or the *UGT1A* rs10929302 A/A genotype). Additionally, the safety analysis was hampered by the low incidence of ADRs and the administration of a single dose to the majority of subjects, who were healthy volunteers.

The available sample size was limited and arbitrary since this is a candidate gene study based on the available pharmacokinetic clinical trial. In addition, the *p*-value was arbitrarily established at <0.05 and was used as the significance threshold, as this is an observational study where the sample size was not calculated to detect a specific effect. Therefore, the results presented here should be considered with caution. For this reason, it would be advisable to increase the sample size to enrich our population with carriers of low-prevalence genotypes, which would improve the statistical power of the study. However, this study has several strengths, such as controlled dietary conditions, avoidance of drug interactions, and other pathologies.

## 4. Materials and Methods

### 4.1. Study Population and Study Design

The participants in this pharmacogenetic study were healthy volunteers who took part in three fesoterodine bioequivalence clinical trials (designated A to C) conducted at the Clinical Trials Unit of Hospital Universitario de La Princesa (UECHUP) in Madrid, Spain, in 2018 (Table 4). All of the trials were open-label, crossover, and randomized, featuring two sequences and two periods, with a wash-out period of at least 7 days between periods, except for clinical trial C, which had no wash-out stage between periods. In each period, the subjects were randomly assigned to receive the dose of either the reference or test formulation, being the opposite one administered in the subsequent period.

Information on the demographic parameters (age, sex, biogeographic origin, weight, height, and BMI), pharmacokinetics, and the occurrence of adverse events (AEs) and ADRs was collected from the clinical trial reports.

The three bioequivalence trials compared 4 and 8 mg fesoterodine test formulations (T) with Toviaz^®^ (Pfizer Europe MA EEIG) (reference formulation, R) at the same dose. In clinical trial A, two formulations of 4 mg fesoterodine prolonged-release tablets (one T and one R) were administered as a single dose. In clinical trial B, two formulations of 8 mg fesoterodine prolonged-release tablets (T and R) were also administered as a single dose. In clinical trial C, two formulations of 8 mg fesoterodine prolonged-release tablets (T and R) were administered daily for 5 days, and the bioequivalence calculations were performed at a steady state.

In both periods of each clinical trial, the volunteers were hospitalized from 10 h before dosing until 36 h after dosing. The formulations were administered orally under fasting conditions (in trials A and C) or under fed conditions (in trial B). In the fed condition, the subjects were provided with a high-fat, high-calorie breakfast, following the European Medicines Agency (EMA) guidelines [15].

The inclusion criteria for all the clinical trials included males or females aged from 18 to 55, with no clinically significant organic or psychic conditions and with normal medical records, vital signs, electrocardiogram, and physical examination and without significant abnormalities in the laboratory analysis. The exclusion criteria included taking any medication two days before the start of the trial, having a body mass index (BMI) outside the 18.5–30.0 range, being pregnant or breastfeeding women, having a history of drug sensitivity, having a positive drug screening, smoking or alcoholism, blood donation in the last month, and participation in another study with investigational drugs in the previous three months.

The three bioequivalence trials were approved by the Spanish Drugs Agency (AEMPS) and the Research Ethics Committee (CEIm) of the Hospital Universitario de La Princesa. The trials were conducted, and the data were handled in accordance with Spanish legislation, the International Council on Harmonization (ICH) guidelines on Good Clinical Practice [16], and the revised Declaration of Helsinki [17]. Every healthy volunteer (*n* = 74) signed the informed consent to participate in the clinical trial, and 39 of them also gave written informed consent to participate in the pharmacogenetic study. The Independent Ethics Board of the Hospital Universitario de La Princesa approved this study on November 23, 2021 (registry number 4627).

### 4.2. Pharmacokinetics Analysis

Twenty blood samples were taken upon hospital admission and additional visits during each period, from pre-dose to 48 h after drug intake. As in the multiple-dose clinical trial (C), only the 24 h steady-state concentration–time curve was considered.

The determination of active metabolite concentrations was outsourced to an external laboratory. The analytical method was based on high-performance liquid chromatography coupled with tandem mass spectrometry (HPLC-MS/MS), with a lower limit of quantification (LLOQ) for 5-HMT of 1 ng/mL, validated according to the EMA guidelines [18].

The professional version of Phoenix WinNonlin (Scientific Consulting, Inc, Cary, NC, USA) was used for the pharmacokinetic analysis. The total area under the curve (AUC_∞_) resulting from the sum of two partial AUCs was used as follows: (a) the AUC from the pre-dose to the last observed concentration point (AUC_0-t_) (obtained by the trapezoidal method) and (b) the AUC from point t to infinity (AUC_t-∞_), calculated as C/k_e_, where C is the last detectable concentration and k_e_ is the slope of the line obtained by linear regression from the points corresponding to the elimination phase of the drug. In trial C, the AUC between dosing intervals (AUCtau) was calculated using the trapezoidal method. The Cl/F was calculated as the dose divided by the AUC_∞_ or AUCtau and weight. The C_max_ (C_max-ss_ in a steady state for study C) and T_max_ were obtained directly from the plasma concentration data. Finally, the T_1/2_ was calculated as –ln2/k_e_. Since the test formulations were demonstrated to be bioequivalent to Toviaz^®^, the arithmetic mean of the pharmacokinetic parameters was calculated for each volunteer.

### 4.3. Safety

AEs were identified by the direct questioning of subjects and spontaneous reporting. The causality of AEs was analyzed according to the algorithm of the Spanish pharmacovigilance system [19]. Only those AEs with a definite, probable, or possible relationship with fesoterodine intake were considered ADRs.

### 4.4. Genotyping

One EDTA-K2 tube was used for DNA extraction from the total peripheral blood. A Maxwell^®^ RSC instrument (Promega, USA) was used for this purpose. After the extraction, the DNA concentration was measured with a Qubit^®^ 4 fluorometer (ThermoFisher, Waltham, MA, USA) and homogenized at 30–70 ng/μL. A QuantStudio 12 K Flex qPCR instrument (Applied Biosystems, ThermoFisher, Waltham, MA, USA) was used for genotyping 120 single nucleotide variants (SNVs) in 33 genes that are involved in drug metabolism and transport with an Open Array Thermal Block using a custom array (Table 5). *CYP2D6* deletion (*5), duplication, and the presence of hybrid structures were analyzed using two TaqMan^®^ copy number variation assays targeting exon 9 (Assay ID: Hs00010001_cn), described in a previously published work [20] and 5′UTR (Assay ID: Hs04078252_cn) (Applied Biosystems, Foster City, CA, USA).

### 4.5. Phenotyping and Haplotyping

Genotype-informed phenotypes for metabolizing enzymes or transporters were inferred according to the Clinical Pharmacogenetics Implementation Consortium (CPIC) or the Dutch Pharmacogenetic Working Group (DPWG) guidelines for the following genes: *CYP2B6* [21], *CYP2C19* [22], *CYP2C9* [23], *CYP2D6* [24], *CYP3A4* [25], *CYP3A5* [26], *CYP4F2* [27], *SLCO1B1* [28], and *UGT1A1* [29]. Phenotypes were classified as ultrarapid, rapid, normal, intermediate, and poor metabolizers (UM, RM, NM, IM, and PM, respectively) for the metabolizing enzymes. For the transporters, the function was classified as increased, normal, decreased, or poor function (IF, NF, DF, or PF, respectively). The *NAT2* alleles and phenotypes were defined according to the *NAT2* Pharmacogenomics Knowledgebase (PharmGKB) Very Important Pharmacogene entry [30]. The *CYP2C8* and *CYP2B6* haplotypes were defined according to PharmVar (https://www.pharmvar.org/genes, accessed on 10 June 2024). Otherwise, the variants were analyzed individually for the remaining genes.

### 4.6. Statistical Analysis

The statistical analysis was performed with SPSS software (version 23, SPSS Inc., Chicago, IL, USA). The AUC (the AUC_∞_ for clinical trials A and B and AUCtau for clinical trial C) and C_max_ were divided by the dose/weight ratio (DW) to correct dose and weight effects on the bioavailability. The pharmacokinetic parameters were analyzed according to sex, biogeographic origin, clinical trial, food conditions, genotypes, phenotypes, and the occurrence of ADRs. For the univariate and multivariate analyses, the significance level was established at *p* < 0.05. The Shapiro–Wilk test was used to check the variable distributions. For those not following a normal distribution, a logarithmic transformation was applied, and normality was re-analyzed.

The statistical tests used for the variables that followed a normal distribution were a *t*-test (variables with two categories) or an ANOVA test (variables with three or more categories). In addition, a Bonferroni post hoc analysis was performed when ANOVA was used. Non-parametric tests were used for those that did not follow a normal distribution. The Mann–Whitney test was used for variables with two categories, and the Kruskal–Wallis test was used for those with three or more categories. Multivariate analysis was performed with the DW-corrected variables by linear regression, including the independent variables that were significant in the univariate analysis (i.e., with univariate *p*-values (*p*_uv_) lower than 0.05). The multivariate *p*-value (*p*_mv_), non-standardized-coefficient (β), and R^2^ are presented for significant associations.

For the analysis of the incidence of ADRs according to sex, race, genotype, haplotype, or phenotype, a Chi^2^ test was used.

## 5. Conclusions

An association between the CYP2D6 phenotype and fesoterodine pharmacokinetics was observed in this study of young, healthy volunteers. Thus, the *CYP2D6* gene could be a useful pharmacogenetic biomarker for the treatment of an OAB with this drug if the clinical relevance of these associations is confirmed in clinical trials with patients. The role of *CYP3A4* as a pharmacogenetic biomarker seems to be less relevant in the case of fesoterodine. *UGT* genes could be involved in fesoterodine metabolism, but further studies are required to better characterize the genetic variations in these genes and their role in fesoterodine pharmacokinetics.

## Figures and Tables

**Table 1:** Demographic characteristics of the volunteers included in this study.

Variable		N	Age (Years)	Height (m)	Weight (kg)	BMI (kg/m^2^)
			Median (Q1–Q3)	Mean (SD)	Mean (SD)	Mean (SD)
Sex	Female	14	29.5 (23.75–32)	1.62 (0.07) *	61.21 (9.07) *	23.39 (2.89) *^1^
	Male	25	26 (22–29)	1.76 (0.06)	78.42 (8.84)	25.41 (2.61)
Biogeographic origin	European	21	24 (21.5–28) *^2^	1.72 (0.08)	72.23 (12.19)	24.08 (2.80)
	Other ^#^	18	29 (27–32.75)	1.68 (0.11)	72.25 (12.43)	25.40 (2.82)
Clinical trial	A	6	22.5 (20.5–24) *^3^	1.74 (0.09)	70.98 (9.43)	23.32 (2.64)
	B	23	28 (25–32)	1.70 (0.10)	71.64 (13.84)	24.64 (2.92)
	C	10	27 (22–32)	1.70 (0.09)	74.37 (9.89)	25.60 (2.72)
Total		39	27 (23–32)	1.71 (0.09)	72.24 (12.14)	24.69 (2.85)

N: number of volunteers; * *p*_uv_ < 0.001 compared to males; *^1^ *p*_uv_ < 0.05 compared to males; *^2^ *p*_uv_ < 0.05 compared to other; *^3^ *p*_uv_ < 0.05 compared to B; ^#^ Other: Latin Americans (17) + Sub-Saharan African (1).

**Table 2:** Pharmacokinetic parameters according to sex, biogeographical origin, clinical trial, and fed conditions.

Variable		N	AUC/DW (h*ng*kg/mL*mg)	C_max_/DW (ng*kg/mL*mg)	T_max_ (h)	T_1/2_ (h)	Cl/F (mL*kg/h)
			Mean (SD)	Mean (SD)	Median (Q25–Q75)	Mean (SD)	Mean (SD)
Sex	Female	14	388.43 (131.03)	40.27 (10.84)	5.63 (4.88–6.00)	5.52 (1.74) *	2928.60 (1113.99)
	Male	25	434.25 (186.60)	45.75 (16.47)	5.50 (4.38–6.00)	6.93 (1.31)	2753.28 (1258.33)
Biogeographic origin	European	21	444.88 (195.17)	44.62 (17.76)	5.50 (4.88–6.00)	6.61 (1.42)	2789.95 (1459.72)
	Other #	18	386.20 (128.93)	42.81 (10.76)	5.50 (4.19–5.81)	6.20 (1.82)	2846.86 (831.81)
Clinical trial	A	6	345.12 (88.17)	30.91 (6.46) *^1^	5.50 (5.25–6.19)	6.99 (0.51)	3109.17 (944.74)
	B	23	387.84 (137.34)	44.04 (13.47)	5.25 (4.00–6.00)	5.64 (1.34) *^2^	2987.13 (1282.32)
	C	10	530.31 (222.35)	50.92 (17.00)	5.50 (4.88–6.13)	7.87 (1.53)	2247.33 (1019.17)
Food conditions	Fasting	16	460.86 (202.06)	43.42 (16.96)	5.50 (5.13–6.00)	7.54 (1.30)	2570.52 (1051.87)
	Fed	23	387.84 (137.34)	44.04 (13.47)	5.25 (4.00–6.00)	5.64 (1.34) *^3^	2987.13 (1282.32)
Total		39	417.80 (168.41)	43.78 (14.78)	5.50 (4.50–6.00)	6.42 (1.61)	2816.22 (1196.59)

Since the test formulations were demonstrated to be bioequivalent to Toviaz^®^, the arithmetic mean of every pharmacokinetic parameter was calculated for each volunteer. N: number of volunteers; AUC: AUC_∞_ for clinical trials A and B or AUCtau for clinical trial C; * *p*_uv_ < 0.05 compared to males; *^1^ *p*_uv_ < 0.05 compared to C; *^2^ *p*_uv_ < 0.001 compared to C; *^3^ *p*_uv_ < 0.001 compared to fasting; ^#^ Other: Latin Americans (17) + Sub-Saharan African (1); Underlined: *p*_mv_ < 0.05.

**Table 3:** Significant associations between genotypes or phenotypes and pharmacokinetic parameters.

Genotype/Phenotype/ Haplotype		N	AUC/DW (h*ng*kg/mL*mg)	C_max_/DW (ng*kg/mL*mg)	T_max_ (h)	T_1/2_(h)	Cl/F (mL/h*Kg)
			Mean (SD)	Mean (SD)	Median (Q25–Q75)	Mean (SD)	Mean (SD)
*CES1* rs2244613	C/A	19	408.48 (153.48)	43.49 (15.07)	5.50 (4.00–6.00)	5.84 (1.41) *	2910.52 (1393.65)
	A/A	20	426.65 (185.03)	44.07 (14.89)	5.50 (5.00–6.00)	6.97 (1.63)	2726.62 (1003.14)
CYP2D6	UM	4	217.98 (64.52) *^1^	31.48 (7.95)	5.88 (4.81–6.00)	4.29 (0.32) *^4^	4908.69 (1348.36) *^6^
	NM	22	376.41 (105.13) *^2^	39.29 (10.75)	5.50 (4.50–6.00)	6.68 (1.59) *^5^	2898.48 (908.01) *^2^
	IM/PM	13	549.32 (185.04)	55.18 (15.68) *^3^	5.00 (3.88–6.00)	6.63 (1.44)	2033.16 (707.55)
CYP3A4	NM	32	432.58 (139.55)	45.46 (12.87)	5.50 (4.31–6.00)	6.44 (1.68)	2587.90 (929.56)
	IM	7	350.24 (269.28) *^7^	36.14 (21.11)	5.50 (5.25–6.00)	6.34 (1.33)	3859.94 (1747.83) *^7^
*UGT1A* rs10929302	G/G	17	422.49 (157.97)	45.31 (15.60)	5.50 (4.13–6.00)	6.46 (1.50)	2777.62 (1201.50)
	G/A	18	445.32 (177.94)	45.00 (14.31)	5.50 (4.88–6.00)	6.55 (1.79)	2528.77 (772.37)
	A/A	3	228.83 (97.36) *^8^	31.63 (12.15)	6.00 (-)	5.18 (1.12)	4884.33 (1828.50) *^9^
*UGT1A3/4* rs2008584	A/A	8	494.72 (120.50)	55.70 (12.42) *^10^	5.13 (4.31–6.19)	6.83 (1.55)	2142.73 (559.10)
	A/G	21	421.63 (195.81)	41.18 (15.34)	5.50 (4.75–5.88)	6.52 (1.66)	2839.93 (1142.10)
	G/G	10	348.20 (112.12)	39.72 (10.92)	5.63 (3.75–6.00)	5.88 (1.57)	3305.21 (1494.52)
*UGT1A4* rs2011425	T/T	32	439.12 (175.16)	45.96 (15.10) *^11^	5.50 (4.56–6.00)	6.36 (1.68)	2694.13 (1213.57)
	T/G	7	320.32 (87.18)	33.85 (8.17)	5.50 (3.75–6.00)	6.67 (1.35)	3374.39 (1008.07)
*UGT2B7* rs7668258	T/T	8	485.94 (195.80)	48.80 (14.97)	4.00 (2.75–6.00)	6.84 (1.40)	2452.77 (1228.87)
	T/C	14	366.15 (145.58)	35.29 (11.64) *^12^	6.00 (5.50–6.50) *^13^	6.37 (1.95)	3260.75 (1474.35)
	C/C	17	428.26 (169.10)	48.42 (14.58)	5.00 (4.38–5.50)	6.26 (1.45)	2621.16 (833.32)
Total		39	417.80 (168.41)	43.78 (14.78)	5.50 (4.50–6.00)	6.42 (1.61)	2816.22 (1196.59)

Since the test formulations were demonstrated to be bioequivalent to Toviaz^®^, the arithmetic mean of every pharmacokinetic parameter was calculated for each volunteer. N: number of volunteers; AUC: AUC_∞_ for clinical trials A and B or AUCtau for clinical trial C; * *p*_uv_ < 0.05 compared to A/A; *^1^ *p*_uv_ < 0.001 compared to IM/PM; *^2^ *p*_uv_ < 0.05 compared to UM and IM/PM; *^3^ *p*_uv_ < 0.05 compared to UM and NM; *^4^ *p*_uv_ < 0.05 compared to NM and IM/PM; *^5^ *p*_uv_ < 0.05 compared to UM; *^6^ *p*_uv_ < 0.001 compared to IM/PM; *^7^ *p*_uv_ < 0.05 compared to NM; *^8^ *p*_uv_ < 0.05 compared to G/G y G/A; *^9^ *p*_uv_ < 0.05 compared to G/A; *^10^ *p*_uv_ < 0.05 compared to A/G; *^11^ *p*_uv_ < 0.05 compared to T/G; *^12^ *p*_uv_ < 0.05 compared to C/C; *^13^ *p*_uv_ < 0.05 compared to T/T y C/C; Underlined: *p*_mv_ < 0.05 in multivariate analysis.

**Table 4:** Characteristics of the clinical trials included in this study.

Internal Code	EudraCT Code	Dose	Type of Study	Sample Size
A	2018-002487-73	4 mg	Single dose— fasting	14
B	2018-003657-25	8 mg	Single dose— fed	36
C	2018-004209-25	8 mg/day 5 days	Multiple dose—fasting	24

**Table 5:** Genotyped SNVs.

Gene	SNV	Gene	SNV	Gene	SNV	Gene	SNVs
*5HT1A*	rs6295	*CYP2C8*	rs10509681	*CYP2D6*	rs35742686	*CYP4F2*	rs3093105
*5HT2A*	rs6311		rs1058930		rs3892097		rs3093153
	rs6314		rs11572080		rs5030655		rs3093200
	rs7997012		rs11572103		rs5030656	*NAT2*	rs1799930
*ABCB1*	rs1045642	*CYP2C9*	rs1057910		rs5030862		rs1799931
	rs2032582		rs1799853		rs5030865		rs1801280
	rs1128503		rs19952363		rs5030867	*SLC19A1*	rs1051266
*ABCC2*	rs2273697		rs28371685		rs59421388	*SLC22A1*	rs12208357
	rs3740066		rs28371686		rs61736512		rs34059508
*ABCC3*	rs4793665		rs7900194		rs72549346		rs628031
*ABCG2*	rs2231142		rs9332131		rs72549347		rs72552763
*CES1*	rs2244613	*CYP2C18*	rs11188059		rs72549353	*SLC22A2*	rs316019
	rs71647871		rs2860840		rs77467110	*SLC28A3*	rs7853758
	rs8192935	*CYP2C19*	rs12248560		rs79292917	*SLCO1B1*	rs11045819
*COMT*	rs13306278		rs12769205	*CYP3A4*	rs2242480		rs2306283
	rs4680		rs17884712		rs2740574		rs34671512
	rs4818		rs28399504		rs28371759		rs37332752
	rs5993883		rs41291556		rs35599367		rs4149056
*CYP1A2*	rs12720461		rs4244285		rs4646438		rs59502379
	rs2069514		rs4986893		rs4986910	*UGT1A*	rs10929302
	rs2069526		rs56337013		rs55785340	*UGT1A1*	rs4148323
	rs2470890		rs72552267		rs55901263		rs887829
	rs72547516		rs72558186		rs55951658	*UGT1A3/4*	rs2008584
	rs762551	*CYP2D6*	rs1065852		rs67666821	*UGT1A4*	rs2011425
*CYP2A6*	rs28399433		rs1135822	*CYP3A43*	rs61469810	*UGT1A6*	rs10445704
*CYP2B6*	rs2279343		rs1135840	*CYP3A5*	rs10264272		rs7592281
	rs28399499		rs16947		rs41303343	*UGT1A8A*	rs1042597
	rs3211371		rs26760831		rs776746	*UGT2B10*	rs61750900
	rs34223104		rs28371706	*CYP4F2*	rs11409932	*UGT2B15*	rs1902023
	rs3745274		rs28371725		rs2108622	*UGT2B7*	rs7668258

## Data Availability

Data belong to the clinical trials’ sponsors and may be accessible upon reasonable request to the corresponding authors.

## Supplementary Materials

The following supporting information can be downloaded at: https://www.mdpi.com/article/10.3390/ph17091236/s1, Table S1. Pharmacokinetic characteristics regarding genotypes or phenotypes in the exploratory step.
