# Supplementary material for: An Investigational Study on the Role of CYP2D6, CYP3A4 and UGTs Genetic Variation on Fesoterodine Pharmacokinetics in Young Healthy Volunteers
Source: Pharmaceuticals (Basel). 2024 Sep 19;17(9):1236. doi: 10.3390/ph17091236 (PMC11435314; doi:10.3390/ph17091236)
Supplement: Supplementary file 1 [file pharmaceuticals-17-01236-s001.zip › pharmaceuticals-3168628-supplementary.pdf]

Supplementary Table S1. Pharmacokinetic characteristics regarding genotypes or phenotypes in the exploratory step.

Genotype, haplotype or phenotype	N	AUC/DW (h*ng*kg/mL*mg)	C_{max}/DW (ng*kg/mL*mg	T_{max} (h)	$T_{1/2}$ (h)	Cl/F (mL*Kg/h)
***5HT1A* rs6295**		*P* = 0.187	*P* = 0.083	*P* = 0.227	*P* = 0.698	*P* = 0.193
C/C	14	482.33 (195.55)	48.62 (14.66)	5.25 (3.50-6.00)	6.13 (1.45)	2418.03 (1024.44)
C/G	19	375.79 (150.22)	39.08 (12.42)	5.50 (5.00-6.00)	6.62 (1.90)	3138.40 (1349.72)
G/G	5	430.10 (116.21)	51.74 (18.31)	5.50 (3.75-5.63)	6.50 (1.05)	2469.21 (665.10)
***5HT2A* rs6311**		*P* = 0.553	*P* = 0.905	*P* =0.443	*P* = 0.107	*P* = 0.547
G/G	14	369.02 (109.19)	42.95 (13.58)	5.13 (3.94-6.00)	6.48 (1.95)	2988.03 (993.49)
G/A	20	451.33 (201.92)	44.81 (16.31)	5.50 (5.00-5.94)	6.73 (1.32)	2656.60 (1126.16)
A/A	5	420.24 (151.83)	42.03 (14.23)	6.00 (4.88-6.88)	5.03 (1.10)	2973.61 (2027.02)
***5HT2A* rs6314**		*P* = 0.683	*P* = 0.685	*P* = 0.261	*P* = 0.634	*P* = 0.682
C/C	32	419.36 (178.12)	43.55 (14.34)	5.50 (5.00-6.00)	6.48 (1.64)	2839.86 (1241.89)
C/T	6	433.28 (120.20)	46.30 (19.16)	4.63 (4.06-5.81)	6.13 (1.69)	2552.48 (1039.69)
***5HT2A* rs7997012**		*P* = 0.182	*P* = 0.114	*P* = 0.372	*P* = 0.398	*P* = 0.181
T/T	7	533.07 (198.92)	52.76 (14.49)	6.00 (5.00-6.00)	7.13 (1.10)	2193.63 (1024.09)
T/C	20	407.17 (179.69)	44.02 (16.12)	5.50 (4.56-6.00)	6.16 (1.57)	2959.33 (1366.67)
C/C	12	368.27 (96.35)	38.16 (10.30)	5.38 (4.13-5.69)	6.43 (1.90)	2940.87 (920.46)
***ABCB1* rs1045642**		*P* = 0.228	*P* = 0.228	*P* = 0.336	*P* = 0.057	*P* =0.225
T/T	12	410.89 (150.87)	45.47 (10.63)	5.13 (3.88-6.00)	5.62 (1.56)	2777.26 (1088.57)
T/C	17	460.81 (189.70)	46.67 (14.17)	5.50 (4.25-5.63)	7.05 (1.35)	2500.73 (893.87)
C/C	10	352.97 (140.47)	36.86 (18.85)	5.63 (5.38-6.00)	6.31 (1.76)	3399.30 (1615.24)
***ABCB1* rs1128503**		*P* = 0.825	*P* = 0.486	*P* = 0.766	*P* = 0.927	*P* = 0.836
T/T	8	398.07 (116.80)	40.38 (19.64)	5.50 (5.13-5.88)	6.53 (1.73)	2769.34 (995.27)
T/C	14	396.58 (140.18)	41.79 (11.28)	5.50 (3.69-6.13)	6.50 (1.75)	2964.09 (1425.07)
C/C	17	444.55 (209.94)	47.02 (15.05)	5.25 (4.38-6.00)	6.30 (1.52)	2716.49 (1134.20)
***ABCB1* rs2032582**		*P* = 0.662	*P* = 0.578	*P* = 0.658	*P* = 0.648	*P* = 0.643
T/T	7	419.09 (108.60)	42.49 (20.22)	5.50 (5.00-6.00)	6.58 (1.87)	2592.91 (930.13)
T/G	14	382.40 (144.39)	40.02 (11.75)	5.50 (3.69-6.13)	6.54 (1.73)	3091.09 (1433.47)

G/G	11	461.34 (178.64)	48.21 (11.78)	5.00 (3.75-6.00)	6.62 (1.37)	2472.88 (940.76)
G/A	7	418.88 (251.14)	45.65 (19.25)	5.75 (5.00-6.00)	5.70 (1.60)	3029.30 (1331.32)
***ABCC2* rs2273697**		*P* = 0.174	*P* = 0.541	*P* = 0.382	*P* = 0.302	*P* = 0.177
G/G	26	392.67 (154.12)	41.99 (13.15)	5.50 (4.44-6.00)	6.24 (1.60)	3006.21 (1318.67)
G/A	9	464.64 (132.85)	45.41 (17.47)	5.50 (4.88-6.25)	6.92 (1.92)	2341.70 (724.40)
***ABCC2* rs3740066**		*P* = 0.904	*P* = 0.683	*P* = 0.715	*P* = 0.406	*P* = 0.913
C/C	15	408.02 (153.51)	42.02 (13.10)	5.75 (4.25-6.00)	6.08 (1.71)	2882.54 (1375.40)
C/T	16	436.52 (195.64)	46.58 (17.62)	5.50 (4.56-6.00)	6.82 (1.70)	2742.91 (1141.74)
T/T	7	397.14 (165.12)	42.77 (12.53)	5.25 (4.50-6.00)	6.13 (1.21)	2895.10 (1157.35)
***ABCC3* rs4793665**		*P* = 0.396	*P* = 0.445	*P* = 0.681	*P* = 0.697	*P* = 0.402
C/C	5	332.71 (147.90)	35.88 (13.54)	5.25 (3.63-5.88)	5.85 (1.31)	3538.79 (1515.93)
C/T	15	431.00 (179.77)	44.43 (14.92)	5.50 (4.25-6.00)	6.45 (1.54)	2660.13 (950.88)
T/T	19	429.77 (166.04)	45.36 (15.07)	5.50 (4.50-6.00)	6.54 (1.77)	2749.29 (1280.15)
ABCG2 rs2231142		*P* = 0.765	*P* = 0.507	*P* = 0.200	*P* = 0.485	*P* = 0.755
C/C	31	400.18 (139.09)	42.09 (13.75)	5.50 (4.50-6.00)	6.49 (1.62)	2877.49 (1199.55)
C/A	6	433.12 (203.26)	46.25 (15.07)	4.75 (3.88-5.63)	5.97 (1.82)	277.31 (1268.16)
***CES1* rs8192935**		*P* = 0.692	*P* = 0.942	*P* = 0.082	*P* = 0.089	*P* = 0.690
T/T	2	320.88 (145.12)	40.20 (2.50)	4.75 (4.50-)	4.17 (0.07)	3489.94 (1577.98)
T/C	27	428.21 (177.36)	44.04 (16.03)	5.50 (4.00-6.00)	6.41 (1.46)	2793.67 (1300.51)
C/C	10	409.07 (155.03)	43.80 (13.30)	5.88 (5.50-6.13)	6.89 (1.86)	2742.33 (879.99)
***COMT* rs13306278**		*P* = 0.123	*P* = 0.688	*P* = 0.716	*P* = 0.854	*P* = 0.122
C/C	29	389.78 (138.46)	43.21 (14.03)	5.50 (4.38-6.00)	6.45 (1.65)	2969.34 (1245.65)
C/T	10	499.04 (224.14)	45.43 (17.51)	5.50 (4.88-6.13)	6.42 (1.61)	2372.15 (961.02)
***COMT* rs4680**		*P* = 0.086	*P* = 0.469	*P* = 0.701	*P* = 0.890	*P* = 0.088
G/G	20	359.26 (140.21)	40.18 (15.79)	5.50 (4.88-6.00)	6.47 (1.50)	3241.71 (1334.03)
G/A	10	475.94 (152.39)	45.23 (12.71)	5.25 (4.44-5.81)	6.47 (2.07)	2306.82 (734.85)
A/A	7	437.10 (135.70)	46.64 (8.15)	4.50 (2.75-6.00)	6.13 (1.57)	2561.96 (999.65)
***COMT* rs4818**		*P* = 0.613	*P* = 0.852	*P* = 0.800	*P* = 0.494	*P* = 0.588
C/C	14	378.22 (141.76)	42.27 (12.37)	5.63 (4.13-6.00)	6.03 (1.60)	3138.16 (1458.23)
C/G	21	420.80 (160.25)	42.57 (15.54)	5.50 (4.88-6.00)	6.69 (1.72)	2728.66 (1037.72)

G/G	2	436.17 (188.64)	48.26 (3.05)	4.00 (2.00-)	6.04 (0.62)	2299.95 (104.04)
***COMT* rs5993883**		*P* = 0.377	*P* = 0.216	*P* = 0.695	*P* = 0.296	*P* = 0.378
T/T	5	355.73 (113.81))	44.07 (11.69)	5.25 (4.38-5.63)	6.23 (2.13)	3080.06 (1026.30)
T/G	20	388.45 (131.91)	40.49 (11.98)	5.50 (4.50-6.00)	6.06 (1.51)	2917.93 (1096.91)
G/G	11	492.84 (229.48)	50.51 (20.12)	5.50 (4.75-6.00)	7.04 (1.68)	2584.48 (1542.44)
***CYP1A2* rs2069514**		*P* = 0.950	*P* = 0.972	*P* = 0.298	*P* = 0.750	*P* = 0.950
G/G	31	411.37 (178.40)	42.53 (15.66)	5.50 (4.50-6.00)	6.41 (1.71)	2922.68 (1295.31)
G/A	4	364.86 (54.59)	41.52 (6.28)	5.63 (4.19-6.50)	5.86 (0.96)	2792.31 (419.27)
A/A	1	418.35 (-)	45.48 (-)	2.75 (-)	5.61 (-)	2391.42 (-)
***CYP1A2* rs2069526**		*P* = 0.265	*P* = 0.653	*P* = 0.530	*P* = 0.822	*P* = 0.255
T/T	33	432.89 (176.58)	44.25 (15.79)	5.50 (4.63-6.00)	6.44 (1.51)	2750.20 (1247.86)
T/G	6	334.76 (78.82)	41.24 (7.46)	5.00 (4.31-5.81)	6.28 (2.24)	3179.31 (853.90)
***CYP1A2* rs2470890**		*P* = 0.110	*P* = 0.613	*P* = 0.399	*P* = 0.721	*P* = 0.111
T/T	7	510.77 (160.84)	48.07 (15.79)	5.50 (2.75-6.00)	6.53 (1.41)	2233.60 (1077.61)
T/C	21	406.26 (168.06)	41.85 (15.86)	5.50 (4.88-6.00)	6.46 (1.73)	2850.33 (1073.24)
C/C	10	340.53 (106.50)	42.34 (10.14)	5.00 (3.94-5.81)	6.00 (1.41)	3306.06 (1384.20)
***CYP1A2* rs762551**		*P* = 0.233	*P* = 0.580	*P* = 0.967	*P* = 0.540	*P* = 0.231
C/C	19	392.20 (184.80)	42.41 (16.34)	5.50 (4.50-6.00)	6.25 (1.53)	3080.24 (1357.71)
C/A	20	442.11 (151.96)	45.08 (13.44)	5.50 (4.31-6.00)	6.58 (1.71)	2565.39 (990.65)
CYP2A6		*P* = 0.287	*P* = 0.226	*P* = 0.474	*P*= 0.476	*P* = 0.265
*1/*1	37	419.97 (170.09)	43.91 (14.71)	5.50 (4.50-6.00)	6.38 (1.64)	2803.86 (1202.16)
*1/*9	1	249.52 (-)	25.54 (-)	6.00 (-)	7.58 (-)	4110.77 (-)
CYP2B6		*P* = 0.815	*P* = 0.554	*P* = 0.295	*P* = 0.200	*P* = 0.820
RM	1	302.95 (-)	45.20 (-)	5.50 (-)	4.82 (-)	3301.05 (-)
NM	12	366.30 (106.40)	40.15 (16.30)	5.50 (4.88-5.75)	6.98 (1.59)	2964.60 (855.60)
IM	17	434.17 (176.08)	45.33 (13.40)	5.00 (4.00-6.00)	6.21 (1.67)	2809.95 (1453.66)
PM	4	393.65 (135.46)	34.96 (10.65)	6.00 (5.63-6.56)	5.20 (1.33)	2920.57 (1461.92)
CYP2C8		*P* = 0.488	*P* = 0.706	*P* = 0.741	*P* = 0.269	*P* = 0.485
*1/*1	22	406.63 (147.51)	44.61 (14.46)	5.50 (4.69-6.00)	6.21 (1.42)	2830.98 (1114.69)
*1/*2	2	349.55 (62.49)	40.61 (3.47)	4.75 (3.75-)	4.58 (1.02)	2936.61 (561.21)

*1/*3	8	386.12 (126.22)	38.45 (10.79)	5.75 (2.75-6.38)	7.17 (1.89)	3016.54 (1564.60)
*1/*4	2	642.89 (196.58)	49.97 (26.66)	5.75 (5.50-)	6.42 (3.70)	1632.05 (498.52)
*3/*3 y *3/*4	4	499.87 (315.14)	50.32 (24.55)	5.25 (3.31-5.50)	7.08 (0.91)	2659.82 (1492.01)
CYP2C9		*P* = 0.938	*P* = 0.730	*P* = 0.267	*P* = 0.857	*P* = 0.936
NM	25	410.49 (163.81)	43.08 (14.98)	5.50 (4.63-6.00)	6.26 (1.75)	2854.34 (1144.42)
IM	10	439.23 (210.64)	44.92 (16.27)	5.50 (2.75-6.00)	6.56 (1.44)	2817.63 (1531.13)
PM	1	424.56 (-)	32.24 (-)	6.75 (-)	6.86 (-)	2370.98 (-)
***CYP2C18* rs11188059**		*P* = 0.681	*P* = 0.665	*P* = 0.542	*P* = 0.167	*P* = 0.678
G/G	26	417.00 (189.97)	42.58 (14.61)	5.50 (3.94-6.00)	6.14 (1.31)	2921.16 (1356.34)
G/A	11	433.47 (120.10)	47.20 (15.27)	5.50 (5.00-6.00)	6.77 (2.13)	2496.84 (712.30)
A/A	2	341.98 (130.76)	40.59 (21.28)	5.75 (5.50-)	8.13 (0.78)	3208.43 (1276.10)
***CYP2C18* rs2860840**		*P* = 0.825	*P* = 0.401	*P* = 0.433	*P* = 0.678	*P* = 0.834
C/C	10	418.87 (182.06)	40.00 (13.78)	5.63 (5.25-6.00)	6.15 (1.72)	2829.18 (1228.01)
C/T	16	443.52 (176.71)	47.46 (16.27)	5.13 (4.50-5.88)	6.37 (1.54)	2583.19 (937.45)
T/T	6	389.71 (102.17)	41.04 (10.20)	5.75 (4.44-6.13)	6.92 (2.07)	2762.28 (819.49)
CYP2C19		*P* = 0.853	*P* = 0.538	*P* = 0.485	*P* = 0.866	*P* = 0.853
RM	6	362.67 (83.79)	36.73 (7.86)	5.50 (4.88-6.25)	6.46 (2.31)	2897.81 (648.53)
NM	19	435.50 (190.27)	44.61 (17.52)	5.50 (5.00-6.00)	6.19 (1.58)	2788.88 (1360.41)
IM	9	433.84 (202.69)	44.72 (14.88)	4.50 (3.63-6.00)	6.53 (1.56)	2886.56 (1465.54)
***CYP3A43* rs61469810**		*P* = 0.761	*P* = 0.715	*P* = 0.564	*P* = 0.839	*P* = 0.733
A/A	36	421.56 (173.03)	44.04 (15.04)	5.50 (4.50-6.00)	6.43 (1.65)	2807.61 (1221.56)
A/-	3	372.69 (107.75)	40.73 (13.32)	6.00 (4.50-)	6.23 (1.17)	2919.51 (1036.25)
CYP3A5		*P* = 0.750	*P* = 0.342	*P* = 0.527	0.707	*P* = 0.766
IM	8	397.56 (149.44)	39.30 (11.86)	5.63 (4.63-6.38)	6.22 (1.23)	2914.61 (1268.79)
PM	31	423.02 (174.86)	44.94 (15.41)	5.50 (4.50-6.00)	6.47 (1.71)	2790.82 (1197.83)
CYPAF2		*P* = 0.316	*P* = 0.288	*P* = 0.897	*P* = 0.899	*P* = 0.315
*1/*1	12	445.17 (141.79)	54.00 (13.40)	5.50 (4.44-6.00)	6.42 (1.42)	2488.08 (857.35)
*1/*3	3	342.82 (109.62)	37.70 (5.13)	4.50 (3.75-)	6.16 (1.70)	3190.15 (1230.99)
*1/*4	11	411.56 (140.08)	42.65 (10.79)	5.50 (4.00-6.50)	6.36 (1.72)	2846.50 (1442.67)
*1/*5	3	307.47 (113.17)	39.12 (11.32)	5.50 (2.00-)	5.27 (1.06)	3591.65 (1386.46)

*1/*6	2	277.44 (39.48)	8.92 (6.31)	5.88 (5.75-)	5.72 (2.63)	3722.10 (549.65)
*1/*2	3	569.61 (324.51)	20.55 (11.86)	5.00 (4.50-)	6.26 (1.11)	2139.25 (962.80)
NAT2		*P* = 0.884	*P* =0.715	*P* = 0.276	*P* = 0.961	*P* = 0.886
**1/*1*	1	338.12 (-)	45.56 (-)	5.75 (-)	6.48 (-)	2957.41 (-)
**1/*5*	10	428.94 (146.33)	40.44 (12.33)	5.75 (5.44-6.56)	6.60 (1.88)	2616.41 (920.05)
**1/*6*	3	334.64 (103.82)	33.47 (11.93)	5.50 (2.75-)	6.87 (1.10)	3239.92 (1178.02)
**1/*7*	2	486.29 (73.33)	54.74 (1.27)	4.13 (2.75-)	7.58 (1.56)	2082.70 (315.91)
**5/*6*	13	409.14 (227.41)	44.68 (20.10)	5.50 (4.63-6.00)	6.31 (1.59)	3186.11 (1638.91)
**5/*7*	1	419.05 (-)	46.22 (-)	4.50 (-)	5.52 (-)	2421.38 (-)
**5/*5*	7	474.90 (150.64)	49.96 (10.75)	4.25 (2.75-5.75)	6.16 (1.87)	2276.74 (623.93)
**6/*6*	1	409.53(-)	32.57 (-)	5.50 (-)	7.18 (-)	2442.01 (-)
***SCL19A1* rs1051266**		*P* = 0.350	*P* = 0.148	*P* = 0.080	*P* = 0.262	*P* = 0.353
A/A	7	383.65 (108.85)	42.23 (11.57)	4.50 (2.75-5.50)	7.12 (1.47)	2815.19 (828.59)
A/G	25	451.61 (191.23)	46.80 (15.97)	5.75 (4.88-6.00)	6.11 (1.74)	2705.12 (1359.88)
G/G	7	331.17 (81.37)	34.58 (9.51)	5.50 (4.50-5.50)	6.84 (1.00)	3213.99 (865.20)
***SCL22A1* rs12208357**		*P* = 0.219	*P* = 0.595	*P* = 0.345	*P* = 0.283	*P* = 0.212
C/C	30	397.40 (153.73)	43.77 (14.40)	5.50 (4.44-6.00)	6.27 (1.60)	2956.52 (1267.41)
C/T	5	479.57 (123.08)	40.08 (12.92)	5.50 (5.13-6.63)	7.15 (2.14)	2211.30 (614.74)
***SCL22A1* rs34059508**		*P* = 0.822	*P* = 0.590	*P* = 0.219	*P* = 0.807	*P* = 0.836
G/G	37	420.57 (172.15)	44.09 (15.03)	5.50 (4.50-6.00)	6.40 (1.63)	2817.96 (1225.87)
G/A	2	366.59 (73.20)	38.20 (10.30)	4.00 (2.75-)	6.70 (1.53)	2783.86 (554.99)
***SCL22A1* rs628031**		*P* = 0.132	*P* = 0.880	*P* = 0.526	*P* = 0.183	*P* = 0.122
A/A	5	477.06 (189.22)	41.69 (15.57)	5.50 (4.25-6.38)	6.48 (1.87)	2357.25 (862.23)
A/G	10	465.38 (165.72)	44.95 (13.23)	5.88 (4.69-6.31)	7.17 (1.30)	2471.87 (1099.38)
G/G	21	359.79 (121.79)	42.47 (14.47)	5.50 (4.38-5.88)	5.99 (1.70)	3185.20 (1269.79)
***SCL22A1* rs72552763**		*P* = 0.413	*P* = 0.747	*P* = 0.598	*P* = 0.531	*P* = 0.396
GAT/GAT	24	441.73 (192.45)	44.40 (14.42)	5.50 (4.50-6.00)	6.28 (1.56)	2738.07 (1302.88)
GAT/-	15	379.50 (116.55)	42.80 (15.82)	5.50 (4.50-6.00)	6.63 (1.73)	2941.24 (1034.62)
***SCL22A2* rs316019**		*P* = 0.958	*P* = 0.836	*P* = 0.865	*P* = 0.220	*P* = 0.962
T/T	1	423.49 (-)	41.97 (-)	5.13 (4.63-5.88)	4.12 (-)	2374.14 (-)

T/G	8	447.43 (248.28)	46.92 (17.33)	5.50 (4.13-6.00)	5.94 (1.51)	2865.21 (1410.65)
G/G	29	409.71 (150.86)	43.37 (14.63)	5.13 (4.75-5.75)	6.60 (1.62)	2830.85 (1197.01)
***SCL28A3* rs7853758**		*P* = 0.365	*P* = 0.704	*P* = 0.281	*P* = 0.107	*P* = 0.355
C/C	22	400.31 (134.54)	41.75 (15.19)	5.50 (4.63-6.00)	6.50 (1.76)	2878.08 (1260.98)
C/T	13	426.23 (194.77)	45.43 (14.61)	5.00 (3.63-5.63)	6.37 (1.08)	2748.89 (1059.37)
T/T	2	261.81 (61.59)	38.29 (0.19)	5.13 (4.50-)	4.04 (0.25)	3969.59 (899.65)
SLCO1B1		*P* = 0.082	*P* = 0.176	*P* = 0.733	*P* = 0.410	*P* = 0.085
Increased function (IF)	2	201.31 (70.22)	24.44 (1.67)	5.75(5.50-)	5.39 (1.18)	5299.77 (1832.02)
Normal function (NF)	28	444.91 (179.81)	46.48 (15.27)	5.50 (4.50-5.94)	6.47 (1.60)	2628.98 (1051.86)
Decreased function (DF)	6	389.43 (104.39)	39.50 (11.28)	5.50 (4.44-6.19)	5.88 (1.39)	2831.51 (1137.74)
Poor function (PF)	2	355.92 (132.36)	38.96 (16.21)	5.75 (5.50-)	7.81 (3.13)	3019.27 (1121.31)
UGT1A1		*P* = 0.206	*P* = 0.420	*P* = 0.475	*P* = 0.182	*P*= 0.208
NM	14	451.92 (154.97)	47.80 (15.67)	5.50 (4.44-6.06)	6.53 (1.59)	2560.53 (1112.60)
IM	18	427.09 (187.21)	43.39 (15.23)	5.50 (4.88-5.81)	6.66 (1.76)	2709.64 (974.38)
*P*M	5	319.04 (143.68)	37.70 (12.00)	6.00 (4.25-6.88)	5.15 (0.88)	3825.51 (1945.67)
***UGT1A6* rs10445704**		*P* = 0.148	*P* = 0.408	*P* = 0.877	*P*= 0.282	*P* = 0.157
G/G	13	420.36 (146.30)	46.12 (16.23)	5.50 (4.38-6.13)	6.54 (1.59)	2722.03 (1080.71)
G/A	16	445.43 (195.80)	45.22 (15.14)	5.50 (4.25-5.88)	6.79 (1.58)	2595.65 (879.38)
A/A	8	319.72 (119.19)	37.55 (12.23)	5.63 (4.63-5.94)	5.70 (1.52)	3671.32 (1681.11)
***UGT1A6* rs7592281**		*P* = 0.454	*P* = 0.972	*P* = 0.616	*P* = 0.840	*P* = 0.467
G/G	34	407.76 (141.77)	42.74 (13.70)	5.50 (4.44-6.00)	6.42 (1.67)	2786.62 (1035.34)
G/T	3	380.15 (252.08)	43.04 (18.64)	5.75 (5.00-)	6.22 (1.56)	3696.98 (2608.30)
***UGT1A8A* rs1042597**		*P* = 0.275	*P* = 0.181	*P* = 0.780	*P* = 0.536	*P* = 0.289
C/C	18	371.59 (130.96)	39.05 (11.59)	5.50 (4.31-6.00)	6.11 (1.60)	3125.73 (1370.87)
C/G	18	465.41 (195.83)	47.96 (17.31)	5.50 (4.94-6.00)	6.66 (1.47)	2499.91 (918.56)
G/G	3	409.34 (165.49)	47.16 (9.46)	4.50 (4.25-)	6.84 (2.74)	2856.92 (1514.52)
***UGT2B10* rs61750900**		*P* = 0.290	*P* = 0.126	*P* = 0.401	*P* = 0.921	*P* = 0.296
G/G	31	429.73 (170.61)	45.63 (15.06)	5.50 (4.50-6.00)	6.41 (1.58)	2687.74 (1011.55)
G/T	8	371.56 (161.70)	36.63 (11.86)	5.63 (4.75-6.38)	6.47 (1.83)	3314.05 (1741.04)
***UGT2B15* rs1902023**		*P* = 0.413	*P* = 0.379	*P* = 0.450	*P* = 0.969	*P* = 0.417

T/T	7	343.47 (124.10)	36.78 (10.31)	5.00 (4.50-5.75)	6.30 (2.11)	3291.53 (1238.78)
T/G	24	435.78 (197.43)	44.88 (17.18)	5.50 (4.81-6.00)	6.42 (1.41)	2811.78 (1329.78)
G/G	8	428.89 (76.93)	46.61 (7.91)	5.50 (4.31-5.69)	6.51 (1.93)	2413.63 (485.84)

The total number of volunteers for some genes is lower than 39 due to errors in the genoty*p*ing technique. All volunteers had wild-ty*p*e genoty*p*es for *CES1* rs71647871, *CYP1A2* rs12720461 and *CYP1A2* rs72547516 and therefore could not be included in the analysis. N: number of volunteers.
